# Low-intensity focused ultrasound targeting the nucleus accumbens as a potential treatment for substance use disorder: safety and feasibility clinical trial

**DOI:** 10.3389/fpsyt.2023.1211566

**Published:** 2023-09-15

**Authors:** James J. Mahoney, Marc W. Haut, Jeffrey Carpenter, Manish Ranjan, Daisy G. Y. Thompson-Lake, Jennifer L. Marton, Wanhong Zheng, James H. Berry, Padma Tirumalai, Ashley Mears, Pierre D’Haese, Victor S. Finomore, Sally L. Hodder, Ali R. Rezai

**Affiliations:** ^1^Department of Behavioral Medicine and Psychiatry, West Virginia University School of Medicine, Rockefeller Neuroscience Institute, Morgantown, WV, United States; ^2^Department of Neuroscience, West Virginia University School of Medicine, Rockefeller Neuroscience Institute, Morgantown, WV, United States; ^3^Department of Neurology, West Virginia University School of Medicine, Rockefeller Neuroscience Institute, Morgantown, WV, United States; ^4^Department of Neuroradiology, West Virginia University School of Medicine, Rockefeller Neuroscience Institute, Morgantown, WV, United States; ^5^Department of Neurosurgery, West Virginia University School of Medicine, Rockefeller Neuroscience Institute, Morgantown, WV, United States; ^6^West Virginia Clinical and Translational Science Institute, West Virginia University School of Medicine, Morgantown, WV, United States

**Keywords:** substance use disorder, focused ultrasound, neuromodulation, craving, addiction

## Abstract

**Introduction:**

While current treatments for substance use disorder (SUD) are beneficial, success rates remain low and treatment outcomes are complicated by co-occurring SUDs, many of which are without available medication treatments. Research involving neuromodulation for SUD has recently gained momentum. This study evaluated two doses (60 and 90 W) of Low Intensity Focused Ultrasound (LIFU), targeting the bilateral nucleus accumbens (NAc), in individuals with SUD.

**Methods:**

Four participants (three male), who were receiving comprehensive outpatient treatment for opioid use disorder at the time of enrollment and who also had a history of excessive non-opioid substance use, completed this pilot study. After confirming eligibility, these participants received 10 min sham LIFU followed by 20 min active LIFU (10 min to left then right NAc). Outcomes were the safety, tolerability, and feasibility during the LIFU procedure and throughout the 90-day follow-up. Outcomes also included the impact of LIFU on cue-induced substance craving, assessed via Visual Analog Scale (VAS), both acutely (pre-, during and post-procedure) and during the 90-day follow-up. Daily craving ratings (without cues) were also obtained for one-week prior to and one-week following LIFU.

**Results:**

Both LIFU doses were safe and well-tolerated based on reported adverse events and MRI scans revealed no structural changes (0 min, 24 h, and 1-week post-procedure). For the two participants receiving “enhanced” (90 W) LIFU, VAS craving ratings revealed active LIFU attenuated craving for participants’ primary substances of choice relative to sham sonication. For these participants, reductions were also noted in daily VAS craving ratings (0 = no craving; 10 = most craving ever) across the week following LIFU relative to pre-LIFU; Participant #3 pre- vs. post-LIFU: opioids (3.6 ± 0.6 vs. 1.9 ± 0.4), heroin (4.2 ± 0.8 vs. 1.9 ± 0.4), methamphetamine (3.2 ± 0.4 vs. 0.0 ± 0.0), cocaine (2.4 ± 0.6 vs. 0.0 ± 0.0), benzodiazepines (2.8 ± 0.5 vs. 0.0 ± 0.0), alcohol (6.0 ± 0.7 vs. 2.7 ± 0.8), and nicotine (5.6 ± 1.5 vs. 3.1 ± 0.7); Participant #4: alcohol (3.5 ± 1.3 vs. 0.0 ± 0.0) and nicotine (5.0 ± 1.8 vs. 1.2 ± 0.8) (all *p*’s < 0.05). Furthermore, relative to screening, longitudinal reductions in cue-induced craving for several substances persisted during the 90-day post-LIFU follow-up evaluation for all participants.

**Discussion:**

In conclusion, LIFU targeting the NAc was safe and acutely reduced substance craving during the LIFU procedure, and potentially had longer-term impact on craving reductions. While early observations are promising, NAc LIFU requires further investigation in a controlled trial to assess the impact on substance craving and ultimately substance use and relapse.

## 1. Introduction

Over 110,000 overdose deaths occurred in 2022, the most in recorded history, and more than 75% of these deaths involved opioids (1). While current treatments, including medication for opioid use disorder (OUD), are beneficial, success rates remain low (2). Treatment outcomes are complicated by co-occurring substance use disorders (SUDs) (3), many of which are without available medication treatments. Research involving neuromodulation for SUD has recently gained momentum. Both noninvasive (e.g., repetitive transcranial magnetic stimulation [TMS] targeting the dorsolateral prefrontal cortex) and invasive (e.g., deep brain stimulation [DBS] targeting subcortical structures such as the nucleus accumbens [NAc]) forms of neuromodulation have shown potential for reducing substance craving and use in individuals with various SUDs (4). However, these neuromodulatory modalities are not without limitations (5, 6). TMS lacks spatial resolution and previously lacked the capability to target deeper brain structures such as the NAc, which plays a key role in reward neurocircuitry (7–9). While a recent form of TMS, referred to as “deep TMS,” has been FDA-approved for smoking cessation and has demonstrated capacity to reach subcortical structures (10–12), it lacks focality and specificity (13, 14). DBS can precisely target subcortical structures, but requires invasive surgery and its possible complications (15).

Magnetic Resonance (MR)-guided transcranial focused ultrasound (FUS) has emerged as a non-invasive technology to target deep structures with precise temporal and spatial resolution. Current FDA approved clinical indications for FUS include essential tremor and Parkinson’s disease which involves ablation at the defined surgical targets using High-Intensity Focused Ultrasound (HIFU) (16). Non-ablative treatment with Low-Intensity Focused Ultrasound (LIFU) is being explored for opening the blood–brain barrier (BBB) and neuromodulation (17–20). LIFU provides a unique capability for precise sub-cortical neuromodulation without the need for surgery or device implantation. The potential utility of LIFU as a treatment for psychiatric disorders is an active area of interest (16, 21) and prior research has demonstrated the capabilities of LIFU in inducing cognitive and/or behavioral changes (22–27). Regarding the mechanism involved in these LIFU-induced behavioral changes, a recent review of the literature (28) noted several studies suggesting that LIFU may be either excitatory or inhibitory and is likely parameter and brain target dependent, thus further investigation is warranted. Given that there have been no published investigations of LIFU for SUD, we initiated this pilot study with the primary goal of evaluating safety, tolerability, and feasibility of LIFU targeting the NAc. Secondary objectives included the assessment of the potential acute (during the LIFU procedure), short-term (7-day), and longitudinal (90-day) impact on substance craving in individuals with SUD.

## 2. Materials and methods

### 2.1. Design

This clinical trial utilized an open-label design to investigate the safety, feasibility, and tolerability of LIFU targeting the NAc in individuals with SUD (Clinicaltrials.gov Identifier: NCT04197921). Secondary outcomes included the acute and longitudinal impact of LIFU on substance craving. This study was approved by the West Virginia University (WVU) Institutional Review Board and approved via an investigational device exemption provided to the device manufacturer (Insightec) by the U.S. Food and Drug Administration (FDA). All participants gave written, informed consent prior to study participation.

### 2.2. Participants

Full inclusion/exclusion criteria are included in the Supplementary information. In brief, participants were required to be 18–60 years, have an OUD diagnosis, and have a negative urine toxicology screen at screening (excluding cannabis). Participants were recruited from WVU’s Comprehensive Opioid Addiction Treatment (COAT) program which utilizes a structured group-based, multidisciplinary, and multimodal approach including behavioral (group and individual therapy) and pharmacological (buprenorphine/naloxone) treatments (29). Since LIFU had not previously been investigated in individuals with SUD, the first two participants had demonstrated COAT program adherence at the time of study enrollment, thereby minimizing risk of attrition (i.e., lost to follow-up) during the 90-day post-LIFU follow-up assessment period. After the first two participants completed follow-up with no LIFU-related/unexpected adverse events (AEs), the FDA approved continuation of the trial with “enhanced” dose (see below) LIFU in two additional participants. Both of these latter participants were less stable in their recovery (i.e., had recent drug use recurrences).

### 2.3. Interventions

Brain MRI was performed on a 3T GE (Architect 3T) and Siemens (Prisma 3T) scanner at baseline, during sonication (as part of the LIFU treatment session), immediately following each sonication, and at designated times following treatment. MRI was used to identify and target the bilateral NAc. Brain MRI sequences included T1 (with/without contrast), T2 fluid-attenuated inversion recovery (FLAIR), T2*, diffusion-weighted (DWI), susceptibility-weighted (SWI), and gradient echo (GRE) imaging.

**Figure 1:**
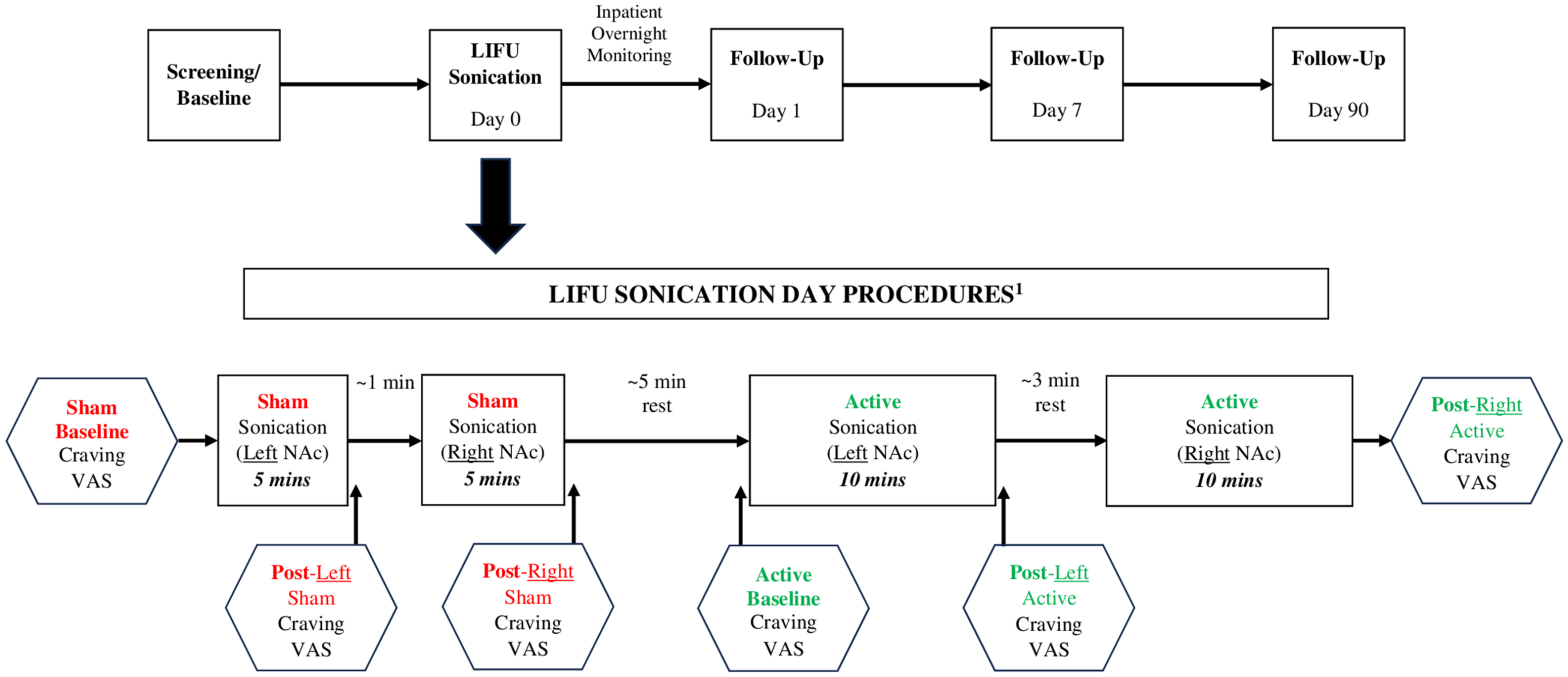
Study schema and LIFU sonication procedures. ^1^Safety Assessment (HAM-D, CSSRS) performed prior to and following Sham and Active LIFU sonications; substance-related cue presentation conducted continuously throughout the course of the entire timeline.

The MR-guided LIFU treatment protocol for blood-brain barrier opening has been previously published by our team (30). Building upon our experience with LIFU, we utilized the ExAblate Neuro Type 2 (Insightec) device/system which utilizes a tranducer helmet array comprised of >1,000 ultrasound transducers which precisely converge on a defined focal point in the brain (NAc) (16). After obtaining baseline assessments (described below), 5 min of single-blinded (participant only) sham sonication was delivered to the left NAc, followed by 5 min to the right NAc. We administered sham LIFU first in all participants as potential carryover effects of active LIFU were unknown given that this was the first known investigation of LIFU in humans with SUD. Next, two 5-min sessions of active sonication were delivered to the left NAc, followed by two 5-min sessions to the right NAc (all participants received the sonications in this order with no hypothesized rationale for sonicating the left NAc first). The first two participants received a 60 W “lower” dose of LIFU at 220 kHz and participants #3 and #4 received a 90 W “enhanced” dose at 220 kHz. Additional sonication parameters are detailed in Table 1. Sham and active sonication procedures were indistinguishable to the participant (e.g., the ultrasound device made the same sound, each session was 5 min, and assessments were conducted identically); however, no energy was delivered during sham sonication. Participants were monitored as inpatients for 24 h post-sonication. The timeline for the assessments/procedures performed on the sonication day is displayed in Figure 1.

**Table 1:** Sham and active LIFU sonication parameters.

	Sham	Lower LIFU dose	Enhanced LIFU dose
Max instantaneous output power (watts)	0	60	90
Intensity (watts/cm^2^)	0	~55	~80
Sonication duration per hemisphere (minutes)	5	10	10
Approximate energy (joules/min)	0	120	180
Repetition time (on/off)	5/10 s		
Pulse duration (on/off)	100/900 ms		
Duty cycle (%)	3.3%		

### 2.4. Assessments

#### 2.4.1. Screening assessments for eligibility determination

Enrolled participants completed behavioral/cognitive assessments, medical, physical and neurological examinations, laboratory assessments, and electrocardiogram during the screening process. Screening procedures/assessments are included in the Supplementary information. Prescreening and eligibility characteristics are included in the Consort Diagram (Supplementary Figure 1).

#### 2.4.2. Safety and tolerability

AEs were assessed prior to, during, and following LIFU sonications and throughout the 90-day follow-up via participant self-report and clinical evaluations which included physical/neurological examinations and behavioral assessments (Hamilton Depression Rating Scale [HAM-D] (31) and Columbia Suicide Severity Rating Scale [CSSRS] (32)). Heart rate was continuously monitored during the LIFU sonication to evaluate any acute physiological changes. Brain MRI scans were acquired to assess for edema, hemorrhage, or any other complications immediately following sonication and at 24 h and 7-day post-procedure follow-up evaluations. Urine toxicology and pregnancy testing were performed during screening, on the day of the LIFU procedure, 24-h post-procedure, and at 7-day and 90-day follow-up assessments.

#### 2.4.3. Cue-induced substance craving (cue reactivity)

Cue reactivity sessions were performed in the following contexts: (a) during screening, (b) prior to, during, and following the sham LIFU sonication, (c) prior to, during, and immediately following the active LIFU sonication, and (d) during each of the follow-up visits. During all cue reactivity sessions participants were exposed to substance-related cues that included images/videos of illicit substances and drug-related paraphernalia. Drug stimuli were individualized to each participant according to their preferred substances, past use, and characteristics of use (e.g., substance type/form, route of administration). Substance craving was assessed using a visual analog scale (VAS) where 0 = “no craving” and 100 = “most craving ever.”

On the day of sonication, the craving VAS was administered at baseline (prior to cue exposure), following 5 min of cue exposure before initiation of LIFU sonication, and at 5-min intervals throughout the sham and active LIFU sonications. Craving ratings were obtained for the three substances (e.g., opioids, heroin, methamphetamine, cocaine, benzodiazepines, cannabis, alcohol, and/or nicotine) which participants preferred/craved the most. Dependent variables included VAS cue-induced craving ratings before, during, and after sham and active LIFU sonications.

During screening and the 7-day and 90-day follow-up evaluations, participants completed a 5-min cue reactivity session (with VAS completed prior to and immediately following cue presentation). The dependent variable included post-cue exposure craving ratings for all the substances mentioned above.

#### 2.4.4. Substance craving outside of the clinic environment (ecological momentary assessment)

The two participants (#3 and #4) who received the enhanced LIFU dose were provided with a phone app (EMA-APP) developed at the Rockefeller Neuroscience Institute (RNI) that prompted them to complete a daily drug craving assessment for one week prior to and one week following the procedure. Craving for substances was assessed via a craving VAS (0 = “no craving”; 10 = “most craving ever”). Dependent variables included average VAS craving ratings across the week preceding and the week following LIFU. The EMA-APP was in development at the time of enrollment for the first two participants who received the lower LIFU dose.

#### 2.4.5. Substance use (urine toxicology and self-report)

Quantitative urine toxicology (gas chromatography–mass spectrometry; GC–MS) was performed during screening and at the 7-day and 90-day follow-ups. Toxicology results were obtained for opioids/opioid analogs, cocaine, amphetamine, benzodiazepines, barbiturates, and delta-9-tetrahydrocannabinol. Self-reported alcohol and substance use was also assessed during screening and all follow-up visits.

### 2.5. Statistical analyses

For all participants, frequency and descriptive analyses were performed for AEs assessed/reported and descriptive analyses were performed for baseline, within-sonication, short term (7-day) and longitudinal (90-day) cue-reactivity VAS craving ratings. For participants #3 and #4, one-way analysis of variance (ANOVA) was used to determine within-subject differences in daily EMA craving ratings in the week pre- and post-LIFU. The *p*-values are descriptive only and have no inferential interpretation as they represent within-subject comparisons of two individual cases. Data were analyzed using SPSS 26.0.

## 3. Results

### 3.1. Study participants

Four participants (three male, one female) were enrolled and successfully completed the study (Table 2). Participants were 30–39 years old, had longstanding histories of OUD, were currently prescribed medication for OUD, and had histories of excessive non-opioid substance use. Participants #3 and #4 (who received the enhanced LIFU dose) reported more recent use of alcohol, cocaine, methamphetamine, and/or benzodiazepines in comparison to participants #1 and #2. All participants reported daily use of ~20 cigarettes.

**Table 2:** Participant demographic and substance use characteristics.

	Lower LIFU dose (60-W)		Enhanced LIFU dose (90-W)	
Participant #	1	2	3	4
Age (years)	39	34	30	33
Gender	M	M	M	F
Substance use (years)				
Opioids	11	13	14	3
Heroin	6	-	3	-
Alcohol	27	18	17	18
Cannabis	27	14	14	11
Methamphetamine	-	1	3	2
Cocaine	7	12	-	14
Benzodiazepines	9	13	14	14
Nicotine	29	9	14	18
Most recent substances used (days since last use)	Cannabis (13)/ Alcohol (>180)	Benzos/Meth/ Cannabis (478)	Alcohol (85)	Alcohol (13)

### 3.2. Safety and tolerability of LIFU procedures

No safety concerns were present during the procedure nor during the 24-h, 7-day and 90-day follow-up evaluations based on clinical observation and subject report. There were no AEs which were unexpected and deemed related to the LIFU procedure and there were no SAE’s (refer to Table 3 for detailed AEs). Behavioral examinations (including the HAM-D and CSSRS) were unremarkable during and immediately following sonications and at follow-up assessments. Brain MRIs acquired immediately following sonication and at 24-h and 1-week post-procedure follow-ups were evaluated by a neuroradiologist and neurosurgeons and did not demonstrate edema, hemorrhage, or notable changes in brain structure.

**Table 3:** Adverse events for all participants through 90-day post-LIFU follow-up.

LIFU Dose	Participant	AE	Severity	Relation	Resolved
Lower Dose (60-W)	1	Anxiety	Moderate	Related to disorder	Yes
		Head pain	Mild	Expected procedure finding	Yes
	2	Scalp swelling	Mild	Expected procedure finding	Yes
Enhanced Dose (90-W)	3	Headache	Mild	Expected procedure finding	Yes
		Nausea	Mild	Expected procedure finding	Yes
		Headache	Mild	Expected procedure finding	Yes
		Insomnia	Mild	Unrelated to device and procedure	Yes
		Hypersomnia	Mild	Unrelated to device and procedure	Yes
		Increased appetite	Mild	Unrelated to device and procedure	Yes
	4	Head pain	Mild	Expected procedure finding	Yes

AEs, adverse events; LIFU, low-intensity focused ultrasound; SUD, substance use disorder; W, watts.

AEs were tabulated during the procedure and follow-up visits for all participants and relatedness to study treatment was established. AEs were deemed “definitely”, “probably”, “possibly”, or “unrelated” to LIFU treatment/procedure and were included in the analysis.

At the conclusion of the study, participants reported a total of 10 AEs throughout the 90-day follow-up (lower LIFU dose: 3 total AEs; enhanced LIFU dose: 7 total AEs), 9 of which were mild and 1 of which was moderate (anxiety in one participant who received the lower LIFU dose deemed not related to the LIFU procedure but related to SUD). Six AEs were expected procedure findings (Lower LIFU dose: 2 procedure-related AEs; enhanced LIFU dose: 4 procedure-related AEs). The most common expected AE was headache/head pain (67%; 4/6). All treatment/procedure-related AEs resolved within 24 h following LIFU completion. Four AEs were deemed unrelated to the treatment/procedure.

### 3.3. Cue-induced substance craving – acute effects of LIFU sonication

*Participants #1 and #2:* VAS craving ratings revealed that there were minimal acute changes in craving following lower dose (60 W) LIFU treatment (Supplementary Figure 2).

**Figure 2:**
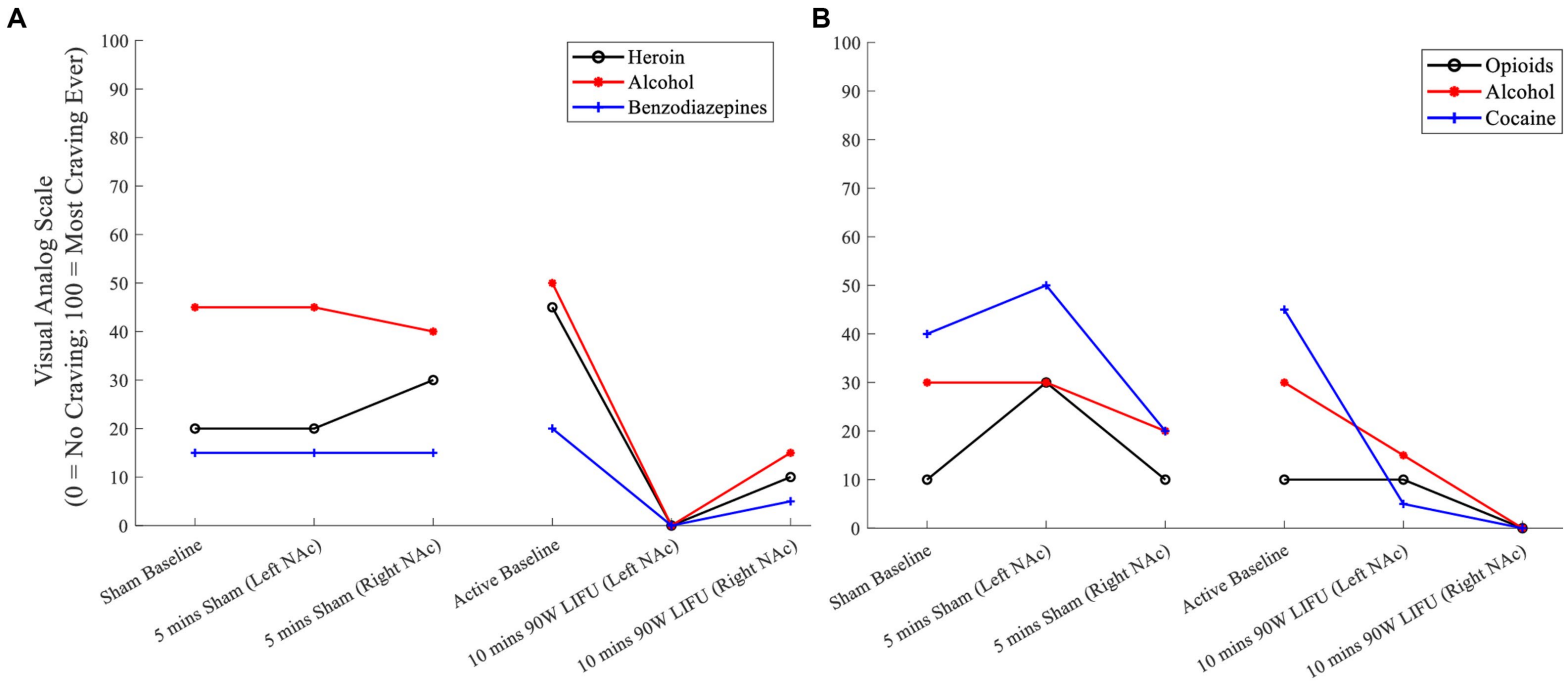
**(A)** Cue-induced substance craving ratings prior to and during Sham and active LIFU (Enhanced Dose) – Participant 3. **(B)** Cue-induced substance craving ratings prior to and during Sham and active LIFU (Enhanced Dose) – Participant 4.

*Participant #3*: VAS craving ratings after 20 min of active dose (90 W) LIFU were attenuated (compared to those ratings after sham sonication) for the participant’s primary substances of choice, including heroin, alcohol, and benzodiazepines (Figure 2A).

*Participant #4*: While there was a reduction in craving during the sham sonication, there was a greater attenuation of craving for the participant’s primary substances of choice, including opioids, alcohol, and cocaine during active LIFU treatment (Figure 2B).

### 3.4. Substance craving (ecological momentary assessment)

*Participants #1 and #2:* EMA was not completed by those who received the lower LIFU dose as the app was under development and therefore, unavailable for use.

**Figure 3:**
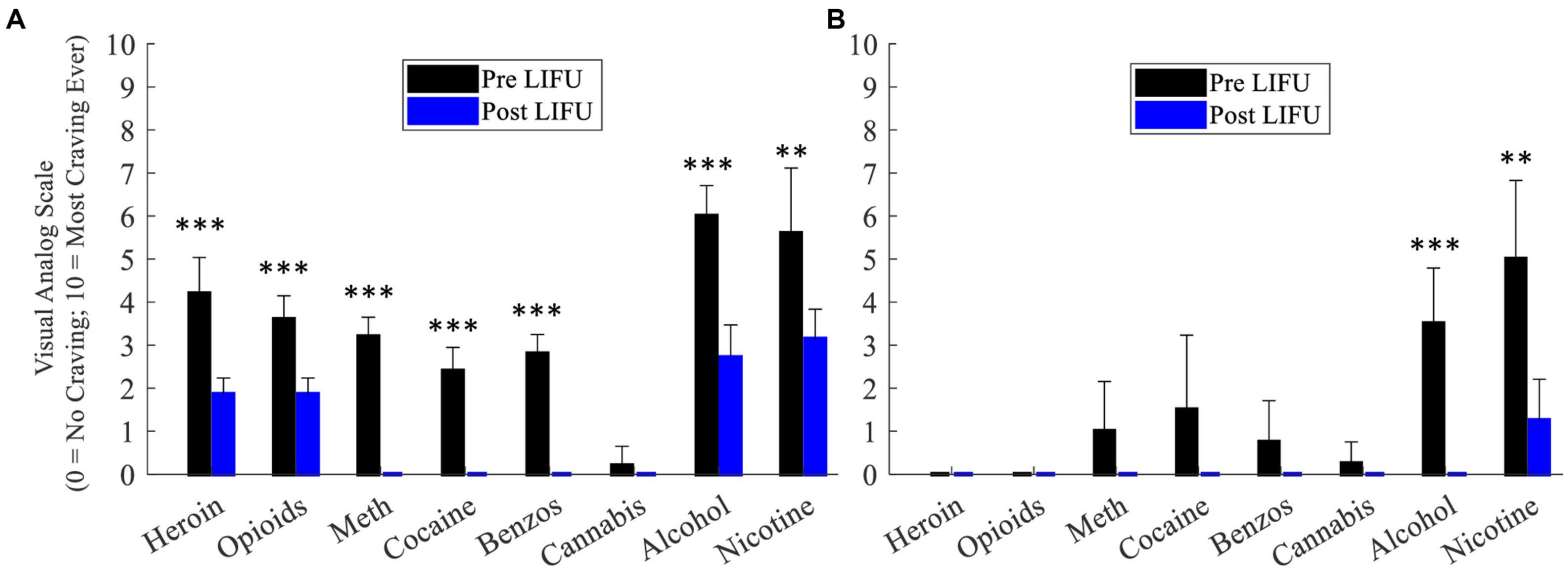
**(A)** Daily craving ratings (week prior to and week following enhanced dose LIFU) – Participant 3. **(B)** Daily craving ratings (week prior to and week following enhanced dose LIFU) – Participant 4. Values represent mean ± standard deviation (M ± SD). *p < 0.05; **p <0.01; ***p < 0.001.

*Participant #3*: Compared to daily EMA VAS craving ratings prior to LIFU, there were significant post-LIFU reductions in craving for the following substances: opioids (3.6 ± 0.6 vs. 1.9 ± 0.4; *F*_1,10_ = 43.07, *p* < 0.001), heroin (4.2 ± 0.8 vs. 1.9 ± 0.4; *F*_1,10_ = 43.78, *p* < 0.001), methamphetamine (3.2 ± 0.4 vs. 0.0 ± 0.0; *F*_1,10_ = 373.33, *p* < 0.001), cocaine (2.4 ± 0.6 vs. 0.0 ± 0.0; *F*_1,10_ = 140.00, *p* < 0.001), benzodiazepines (2.8 ± 0.5 vs. 0.0 ± 0.0; *F*_1,10_ = 285.83, *p* < 0.001), alcohol (6.0 ± 0.7 vs. 2.7 ± 0.8; *F*_1,10_ = 58.00, *p* < 0.001), and nicotine (5.6 ± 1.5 vs. 3.1 ± 0.7; *F*_1,11_ = 14.61, *p* = 0.003; Figure 3A).

*Participant #4*: Compared to daily EMA VAS craving ratings prior to LIFU, there were significant post-LIFU reductions in craving for the following substances: alcohol (3.5 ± 1.3 vs. 0.0 ± 0.0; *F*_1,7_ = 38.11, *p* < 0.001) and nicotine (5.0 ± 1.8 vs. 1.2 ± 0.8; *F*_1,7_ = 17.55, *p* = 0.004). Of note, while pre-LIFU craving ratings were low for all other substances, post-LIFU ratings were consistently rated as “0” and reduced relative to baseline (Figure 3B).

### 3.5. Cue-induced substance craving – prolonged effects of LIFU sonication

**Figure 4:**
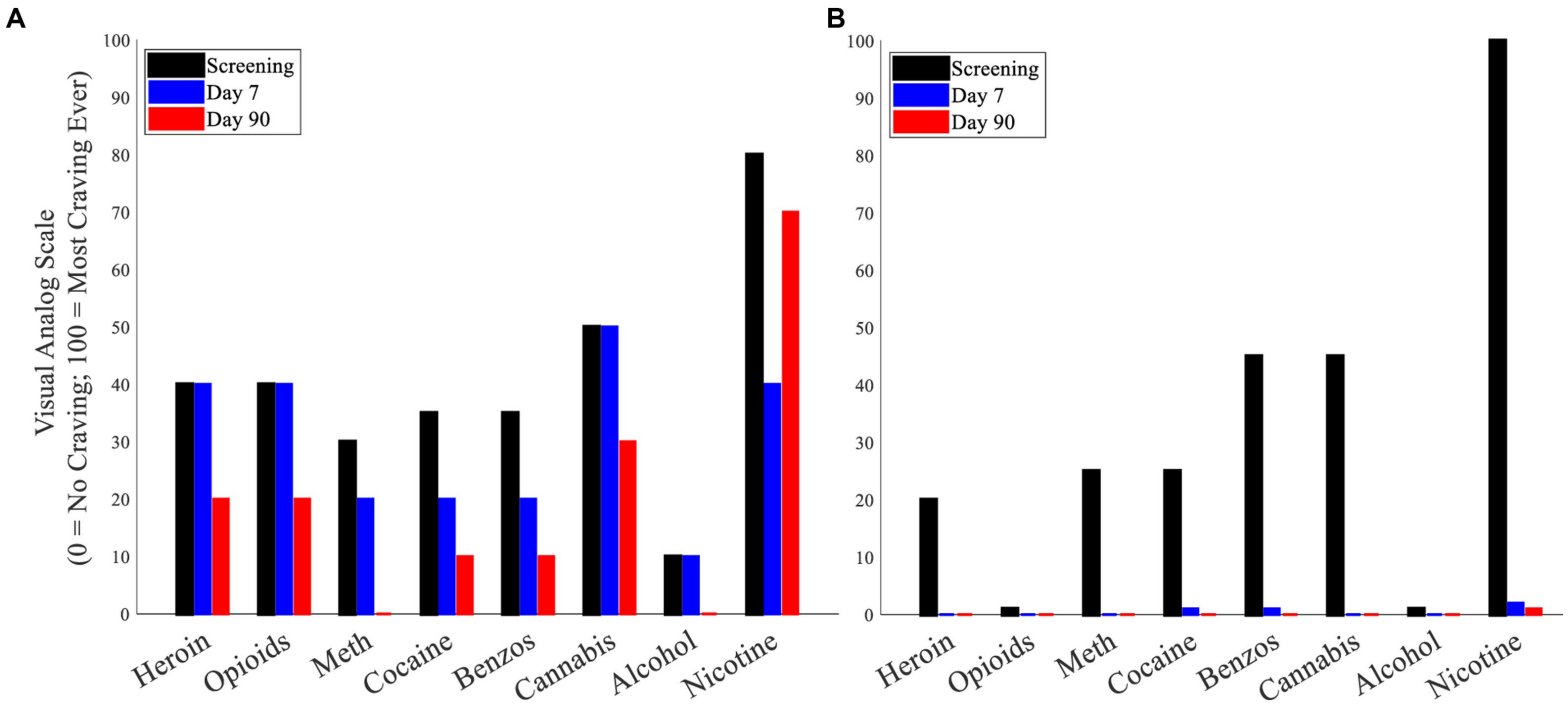
**(A)** Cue-induced substance craving (maximum craving following cue presentation) – Participant 3. **(B)** Cue-induced substance craving (maximum craving following cue presentation) – Participant 4.

Compared to the maximum VAS craving ratings following cue exposure during screening (pre-LIFU), maximum VAS craving ratings at the 90-day post-LIFU follow-up were reduced for several substances in participants who received lower (Supplementary Figure 3) and enhanced LIFU doses (Figure 4).

### 3.6. Urine toxicology and self-reported alcohol and substance use

Participant #1 self-reported regular (weekly) cannabis use throughout enrollment and was positive for cannabis during the Day 7 and Day 90 follow-up visits. Of relevance, he had stated that while he was motivated to abstain from all other illicit drugs, he was not attempting to reduce his cannabis use during the time of study enrollment. He denied the use of any other illicit drugs during enrollment and urine toxicology was negative for all substances other than prescribed buprenorphine. Participants #2, #3, and #4 denied the use of illicit substances during enrollment and urine toxicology during the Day 7 and Day 90 follow-up visits were negative for all substances other than prescribed buprenorphine. Participant #3 and #4 reported that they each had one isolated use of alcohol, but not to intoxication (1–2 beers).

## 4. Discussion

This proof-of-concept study demonstrates that NAc LIFU sonications among persons with SUD is safe, feasible, and well-tolerated. Additionally, for the two participants receiving the enhanced LIFU dose (90 W), cue-induced craving for several substances decreased acutely and remained decreased for 90 days after receiving one LIFU sonication. These data are consistent with the known critical role of the NAc in addiction and supported by published literature reporting reduced substance use and craving with DBS of NAc. However, LIFU may offer significant advantages as it is a non-invasive method of neuromodulation that can precisely target and modulate the subcortical NAc. It is of interest that the short duration of NAc sonication not only acutely reduced cravings, but may have also resulted in sustained craving reduction. Compared to craving ratings in the days preceding the LIFU procedure, craving ratings were lower across the week following sonication for the two participants receiving the enhanced LIFU dose. Furthermore, relative to screening, longitudinal reductions in cue-induced craving for several substances persisted during the 90-day post-LIFU follow-up evaluation for all participants. A caveat to these findings is that they reflect single time-point comparisons (screening versus 90-day follow-up), thus they are susceptible to state-dependent fluctuations in craving dependent on other factors (e.g., psychosocial stress, mood) at the time of assessment. Also, these reductions may reflect the normal course of craving reductions independent from LIFU given the participants continued engagement in treatment.

During the follow-up assessments, the participants who received the enhanced LIFU dose subjectively described reduced substance cravings (both generally and during exposure to triggers in their environment), improvements in overall mood, and reductions in anxiety. It is also important to note that none of the participants reported a decrease in craving/desire for natural reinforcers such as food, water, and social interaction. In addition, both participants reported that when viewing the substance related cues during the active sonication, they had difficulty “connecting” with the cues which was not the case during the sham sonication. In addition to the reductions in craving, these two participants denied illicit substance use throughout the course of the study and each participant reported an isolated event of alcohol use although not to intoxication. Of note, one of these participants reported that the alcohol use was not secondary to increased craving but rather related to “fitting in” during a social interaction.

This is the first investigation of LIFU in individuals with SUD. Enhanced dose NAc LIFU sonication appears to be promising with “therapeutic” response; however, the results must be interpreted in the context of the following limitations. This report includes behavioral data for two participants who received the enhanced dose of LIFU and these findings must be validated with additional participants in a randomized, controlled trial utilizing a “sham-only” design to account for potential (over)exposure effects during the cue reactivity task. As mentioned previously, one participant had a craving reduction during the initial sham sonication. While this must be considered when interpreting the craving reduction following the active dose, we do believe that the active treatment had an independent effect based on the complete suppression of craving during the active sonication. As further evidence, this participant self-reported “difficulty connecting with the cues” during the active LIFU sonication which was similar to the report of the other participant who received the enhanced LIFU dose. In addition, we opted to consistently administer sham LIFU first as we were unsure of potential carryover effects (and duration) of active LIFU given this was the first known investigation of LIFU in individuals with SUD. Future trials should randomize the order of sham and active sonications. Since the primary endpoints for the current pilot study were safety and feasibility, future trials should include a longer duration of follow-up with a rigorous longitudinal assessment of craving and mood, thereby providing a more conclusive determination of the prolonged effects produced by LIFU. Given the subjective nature of the craving VAS, future studies should also incorporate objective measures to evaluate biological measures which may be associated with craving such as heart rate (assessed via wearables technologies) and stress (assessed via salivary cortisol).

In conclusion, LIFU treatment of persons with SUD was safe. Enhanced LIFU doses (90 W) resulted in a “therapeutic” response with subjective improvements in mood and decreased substance cravings. We believe that non-invasive LIFU targeted to the NAc deserves further investigation. Specifically, efficacy needs to be rigorously studied through randomized, double-blind, sham-controlled trials with assessment of the duration of LIFU effect on substance use.

## Data availability statement

The raw data supporting the conclusions of this article will be made available by the authors, without undue reservation.

## Ethics statement

The studies involving humans were approved by West Virginia University School of Medicine. The studies were conducted in accordance with the local legislation and institutional requirements. The participants provided their written informed consent to participate in this study.

## Author contributions

JJM: conceptualization, methodology, formal analysis, investigation, roles/writing – original draft, supervision, visualization, and data curation. MH: conceptualization, methodology, investigation, and writing – review and editing. JC, WZ, JB, MR, PT, and AM: investigation, and writing – review and editing. JLM: investigation, writing – review and editing, data curation, and project administration. DT-L: visualization, and writing – review and editing. SH: writing – review and editing. PD’H: methodology, and writing – review and editing. VF: methodology, resources, writing – review and editing, and supervision. AR: conceptualization, methodology, investigation, resources, and writing – review and editing, supervision. All co-authors had full access to the study data, edited and agreed upon the final manuscript, and shared the final responsibility for the decision to submit for publication.

## Funding

This research did not receive any specific grant funding from agencies in the public, commercial, or not-for-profit sectors. JJM was supported by the National Institute of General Medical Sciences of the National Institutes of Health under Grant No. U54GM104942. The content is solely the responsibility authors and does not necessarily represent the official views of the National Institutes of Health.

## Conflict of interest

The authors declare that the research was conducted in the absence of any commercial or financial relationships that could be construed as a potential conflict of interest.

## Publisher’s note

All claims expressed in this article are solely those of the authors and do not necessarily represent those of their affiliated organizations, or those of the publisher, the editors and the reviewers. Any product that may be evaluated in this article, or claim that may be made by its manufacturer, is not guaranteed or endorsed by the publisher.

## Abbreviations

Adverse Event: AE
Columbia Suicide Severity Rating Scale: CSSRS
Comprehensive Opioid Addiction Treatment: COAT
Deep Brain Stimulation: DBS
Diffusion-Weighted Imaging: DWI
Ecological Momentary Assessment: EMA
Fluid-Attenuated Inversion Recovery: FLAIR
Food and Drug Administration: FDA
Gradient Echo: GRE
Hamilton Depression Inventory: HAM-D
Low-Intensity Focused Ultrasound: LIFU
Magnetic Resonance Imaging: MRI
Nucleus Accumbens: NAc
Opioid Use Disorder: OUD
Substance Use Disorder: SUD
Susceptibility-Weighted Imaging: SWI
Transcranial Magnetic Stimulation: TMS
Visual Analog Scale: VAS
West Virginia University: WVU
