# Supplementary material for: Low-intensity focused ultrasound targeting the nucleus accumbens as a potential treatment for substance use disorder: safety and feasibility clinical trial
Source: Front Psychiatry. 2023 Sep 15;14:1211566. doi: 10.3389/fpsyt.2023.1211566 (PMC10540197; doi:10.3389/fpsyt.2023.1211566)
Supplement: Supplementary file 1 [file Data_Sheet_1.docx]

**Supplementary Information**

**Screening Procedures and Assessments for Eligibility Determination**

Participants completed intake questionnaires to gather information pertaining to demographic and drug use characteristics (i.e., years of substance use, recent substance use, age of first use, route of administration) for several substances, including opioids, heroin, methamphetamine, cocaine, benzodiazepines, cannabis, alcohol, and nicotine. The SCID-5 was used to assess psychiatric and substance use disorder diagnoses, and the SCID-5-PD was used to assess for underlying personality disorders. The DRS-2 was used to identify cognitive impairment (also repeated 24 hours post-procedure and at 7-day and 90-day follow-up). A medical history, physical examination, and neurological examination were completed, and an electrocardiogram, brain computed tomography (CT) scan, and brain MRI were performed (CT and MRI were also performed within an hour of low-intensity focused ultrasound (LIFU) sonication, 24-hours post-LIFU, and at 7-day follow-up). Laboratory assessments included complete blood count, PT, PTT, aPTT, INR, serum chemistry 7 panel (Na, K, Cl, HCO_3_, blood urea nitrogen, creatinine, glucose), thyroid stimulating hormone, vitamin B12, and glomerular filtration rate. Urine toxicology and a urine pregnancy test (if applicable) were performed (repeated 24 hours post-procedure and at 7-day and 90-day follow-up).

**Inclusion/Exclusion Criteria**

**Inclusion Criteria**

1. Males and non-pregnant females, age 18-60 years old
2. Subject meets DSM-5 criteria for opioid-use disorder (OUD) (assessed via the Structured Clinical Interview for DSM-5 Axis I Disorders [SCID-5]) of at least two years duration
3. Subject is currently receiving outpatient treatment from the WVU Comprehensive Opioid Addiction Treatment Program (COAT), residential treatment from the WVU Center for Hope and Healing, or any other program which implements the COAT model or similar programs that are well known to the research team. The subject will be on a stable dose of medication for opioid use disorder (MOUD; e.g., buprenorphine-naloxone, naltrexone) for the 7 days prior to the procedure. Stable is defined as within the therapeutic range but does not require same exact dose for 7 days.
4. Subject has been off opioids and other illicit substances, except for cannabis; confirmed via urine toxicology screen
5. The nucleus accumbens (NAc) is apparent on magnetic resonsnace imaging (MRI), such that treatment targeting can be performed directly (visible on MRI) and indirectly (using other anatomical structures for measurements)
6. Subject is able to communicate sensations during the Exablate Transcranial procedure
7. Subject is willing to cooperate with the study requirements, including compliance with the regimen and completion of all study visits
8. Subject is able to make own medical decisions as determined by the clinical team
9. Subject has signed and received a copy of the approved informed consent form

**Exclusion Criteria**

Subjects who meet any of the following exclusion criteria must be excluded from the clinical investigation:

1. Subjects who are taking prohibited medications that may adversely interact with MOUD. Being on one of these medications would not automatically exclude a participant from study participation and will be determined at the discretion of a study investigator.
2. Subject with standard contraindications for MRI, such as non-MRI compatible implanted metallic devices
3. Subject with known intolerance or allergies to the MRI contrast agent gadolinium (GADOVIST^®^)
4. Subject who are unable or unwilling to tolerate the required prolonged stationary position during treatment (approximately 2-3 hours)
5. More than 30% of the skull area traversed by the sonication pathway is covered by scars, scalp disorders (e.g., eczema), or atrophy of the scalp
6. Subject with implanted objects in the skull or the brain
7. Subject diagnosed with advanced kidney disease or on dialysis
8. Subject with impaired renal function with estimated glomerular filtration rate < 30 mL/min/1.73 m^2^
9. Subject with known unstable cardiac status or severe hypertension, including:
   1. Documented myocardial infarction within six months of enrollment
   2. Unstable angina on medication
   3. Unstable or worsening congestive heart failure
   4. Left ventricular ejection fraction below the lower limit of normal
   5. History of a hemodynamically unstable cardiac arrhythmia
   6. Cardiac pacemaker
   7. Severe hypertension (diastolic blood pressure > 100 on medication)
10. Subject with history of abnormal bleeding, hemorrhage, or coagulopathy
11. Subject receiving anticoagulant (e.g., warfarin) or antiplatelet (e.g., aspirin) therapy within one week of focused ultrasound procedure or drugs known to increase risk or hemorrhage (e.g., Avastin) within one month of focused ultrasound procedure
12. Abnormal coagulation profile (platelets < 100,000/μl), prothrombin time (PT) (> 13.9 sec) or partial thromboplastin time (PTT; aPTT) (> 37.5 sec) and international normalized ratio (INR) > 1.2. If values are outside the range of normal limits, any clinically significant value may be excluded as determined by a study investigator.
13. Subject with cerebrovascular disease as determined by MRI according to the Fazekas criteria. Grades II and III on the Fazekas scale should be excluded.
14. Past or present diagnosis of schizophrenia, psychotic disorder, bipolar disorder, or untreated depression other than one determined to be substance-induced (assessed via SCID-5)
15. Score of greater than 17 on the Hamilton Depression Rating Scale or increased risk of suicide based on any positive response regarding passive or active suicidal ideation with or without intent over the past 3 months or lifetime history of active suicidal ideation with intent on the Columbia-Suicide Severity Rating Scale at baseline
16. History of suicide attempt
17. Parental history of completed suicide
18. Subject met the criteria for Cluster A or B Personality Disorders (assessed via Structured Clinical Interview for DSM-5 Axis II Personality Disorders [SCID-5-PD])
19. Diagnosis of dementia or any other disorder that has led to a clinically significant cognitive impairment (assessed via Dementia Rating Scale-2 [DRS-2])
20. Current comorbid substance use disorder of alcohol or sedative/hypnotic/anxiolytic based on DSM-5 criteria at a mild level assessed via the SCID-5. Tobacco use disorder, cannabis use disorder, stimulant use disorder, and hallucinogen use disorder are allowed, but OUD must be the primary disorder and the other use disorders must occur in the context of not consistent use.
21. Subject with brain tumors
22. Subject with chronic pulmonary disorders (e.g., severe emphysema, pulmonary vasculitis, or other causes of reduced pulmonary vascular cross-sectional area)
23. Any known central nervous system infection or infection with human immunodeficiency virus or hepatitis C virus (HCV). A diagnosis of HCV infection alone is not exclusionary as long as hepatic function laboratory values are deemed not clinically significant by a study investigator and are ≤ 1.5 times the upper limit of normal.
24. Subject has had deep brain stimulation or a prior stereotactic ablation of the NAc, basal ganglia, or thalamus
25. Subject has been administered botulinum toxins into the arm, neck, or face for 5 months prior to baseline
26. Subject is currently participating in another clinical investigation with an active treatment arm
27. Subject unwilling to abstain from illicit substance use during the course of the study
28. Subject is considered a poor surgical or study candidate, which may include but is not limited to the following: any medical, social, or psychological problem that could complicate the required procedures and evaluations of the study in the judgment of the investigator
29. Subject is non-English speaking
30. Subject is pregnant or planning to become pregnant

**Supplementary Figure 1.** Pre-screening, Eligibility, Enrollment, and Analyses

Pre-Screening

Assessed for Eligibility (n = 260)

Excluded (n = 217)

- Not meeting inclusion criteria (n = 195)
- Unable to determine eligibility based on available information (n = 22)

Met Preliminary Eligibility Criteria

(n = 43)

Excluded (n = 33)

- Unable to contact/Lost contact with patient (n = 21)
- Patient declined participation (n = 12)

Consented (n = 10)

Excluded (n = 5)

- Screen Failure (n = 5)
- Could not tolerate the MRI due to feeling claustrophobic (n=3), abnormal bloodwork (n=1), unable to be contacted to complete screening (n=1)

Eligible/Enrolled (n = 5)

Excluded (n = 1)

- Patient eligible following screening but unable to be contacted to complete procedure (n = 1)

Analyzed (n = 4)

Details regarding how many patients were pre-screened, how many met/did not meet prescreening eligibility criteria, how many who met pre-screening eligibility were consented and enrolled. The most common reasons for individuals not meeting pre-screening eligibility criteria were the following: 1) history of suicide attempt; b) severe comorbid psychiatric diagnosis; 3) abnormal recent laboratory bloodwork; 4) history of seizures. For those who met pre-screening criteria (n=43), twenty-one patients were unable to be contacted and twelve reported that they were not interested and therefore did not consent. Of the ten patients who consented, five were screen failures (could not tolerate the MRI due to feeling claustrophobic (n=3), abnormal bloodwork, unable to be contacted to complete screening) and of the five eligible, one patient was unable to be contacted once deemed eligible following screening completion. The four remaining completed all protocol requirements and procedures and the data from these four patients are presented in the current manuscript.

**Supplementary Figure 2a.** Cue-Induced Substance Craving Ratings Prior to and During Sham and Active LIFU (Lower Dose) – Participant 1

**Supplementary Figure 2b.** Cue-Induced Substance Craving Ratings Prior to and During Sham and Active LIFU (Lower Dose) – Participant 2

**Supplementary Figure 3a.** Cue-Induced Substance Craving (Maximum Craving Following Cue Presentation) – Participant 1

**Supplementary Figure 3b.** Cue-Induced Substance Craving (Maximum Craving Following Cue Presentation) – Participant 2
